# Pro-cancerogenic effects of spontaneous and drug-induced senescence of ovarian cancer cells in vitro and in vivo: a comparative analysis

**DOI:** 10.1186/s13048-022-01023-y

**Published:** 2022-07-26

**Authors:** Szymon Rutecki, Paulina Szulc, Martyna Pakuła, Paweł Uruski, Artur Radziemski, Eryk Naumowicz, Rafał Moszyński, Andrzej Tykarski, Justyna Mikuła-Pietrasik, Krzysztof Książek

**Affiliations:** 1 Department of Pathophysiology of Ageing and Civilization Diseases, Poznań University of Medical Sciences, Długa 1/2 Str, 61-848 Poznań, Poland; 2 Department of Hypertensiology, Poznań University of Medical Sciences, Długa 1/2 Str., 61-848 Poznań, Poland; 3 General Surgery Ward, Medical Centre HCP, 28 czerwca 1956 r. 223/229 Str., 61-485 Poznań, Poland; 4 Division of Gynecological Surgery, Poznań University of Medical Sciences, Polna 33 Str, 60-535 Poznań, Poland

**Keywords:** Drug-induced senescence, Ovarian cancer, Senescence-associated secretory phenotype, Spontaneous senescence

## Abstract

**Background:**

Clinical outcomes of cancer cell senescence are still elusive. Here, we reveal and compare pro-cancerous activity of spontaneously and drug-inducible senescent ovarian cancer cells. Experiments were performed on tumors and tumor-derived primary epithelial ovarian cancer cells (pEOCs) that were obtained from chemotherapy-naïve patients and from patients who received carboplatin (CPT) and paclitaxel (PCT) before cytoreduction.

**Results:**

The analysis of tumors showed that senescent cancer cells are present in patients from both groups, albeit most frequently and covering a greater area in tissues from chemotherapy-positive women. This in vivo senescence of pEOCs translated to an expression of senescence markers in early-passage cells in vitro. A conditioned medium from senescent pEOCs fueled the cancer progression, including adhesion of non-senescent pEOCs to normal peritoneal cells, and their increased proliferation, migration, invasion, and EMT. Senescent pEOCs’ secretome promoted angiogenic activity of vascular endothelium, induced senescence of normal peritoneal cells, reprogrammed their secretome towards hypersecretion of cancer-promoting proteins, and stimulated motility of cancer cells subjected to a mesothelium- and fibroblast-derived medium. The most striking finding was, however, that spontaneously senescent pEOCs supported all the above pro-cancerous effects more efficiently than drug-inducible senescent cells, which was plausibly related to augmented release of several cancer spread mediators by these cells. The prevalence of spontaneously senescent pEOCs was most evident in experiments on mice when they were able, unlike the drug-inducible cells, to promote the development of drug-sensitive i.p. xenografts.

**Conclusions:**

Our study shows that spontaneous senescence of pEOCs should be treated as an independent pathogenetic factor of cancer progression.

## Background

Cellular senescence is considered to be an anti-cancer phenomenon because, *per* definition, senescent cells irreversibly lose their ability to replicate and thus are resistant to neoplastic conversion [1]. At the same time, and to some extent paradoxically, the senescent cells effectively support the expansion of cancer cells in vitro and in vivo [2], which is causatively linked with the so-called senescence-associated secretory phenotype (SASP) [3]. This rule applies to the pathophysiology of ovarian cancer whose progression in culture models and laboratory animals was promoted by senescent normal peritoneal mesothelial cells (PMCs) and whose rejuvenation by targeting p38 MAPK translated to reduced tumor growth [4].

As per senescence of cancer cells, the mechanisms and the clinical significance of this process are far less understood [5]. There is a consensus that cancer cells may be forced to senescence by radio- and chemotherapy [6], and a growing body of evidence suggests that the spontaneous variant of senescence, that is the process occurring in oncologic patients who had not received chemotherapy, is something more than a negligible artifact [7]. The interpretation of the role of senescent cancer cells is difficult because, at least theoretically, their presence may give rise to both positive and negative consequences for a patient [5]. The positive effect is associated with growth inhibition of targeted cells, plausibly restricting disease progression. In turn, the adverse outcome is driven by SASP that exhibit senescent cancer cells similarly to normal cells [8]. There is also an important question of whether the outcomes of spontaneously and drug-inducible senescent cancer cells are identical (in terms of their scale and direction) or maybe different?

Epithelial ovarian cancer (EOC) is one of the most common and the most lethal malignancy of female genital tract [9]. Recent studies have documented the incidence and molecular mechanisms of spontaneous senescence of primary EOC cells (pEOCs) [10]. This was followed by a demonstration of a distinct mechanism underlying senescence of pEOCs triggered by carboplatin (CPT) and paclitaxel (PCT) [11], being the first line chemotherapy for EOC patients [12]. Having this knowledge, we designed a project to answer questions of a more practical nature: i) What is the frequency of senescent EOC cells in tumors from chemotherapy-naïve patients and from patients who received CPT and PCT prior cytoreduction? and ii) What is the effect of both kinds of pEOC senescence on the progression of proliferating cancer cells in vitro and in vivo?

## Results

### Senescent cancer cells in ovarian tumors in vivo

**Fig. 1:**
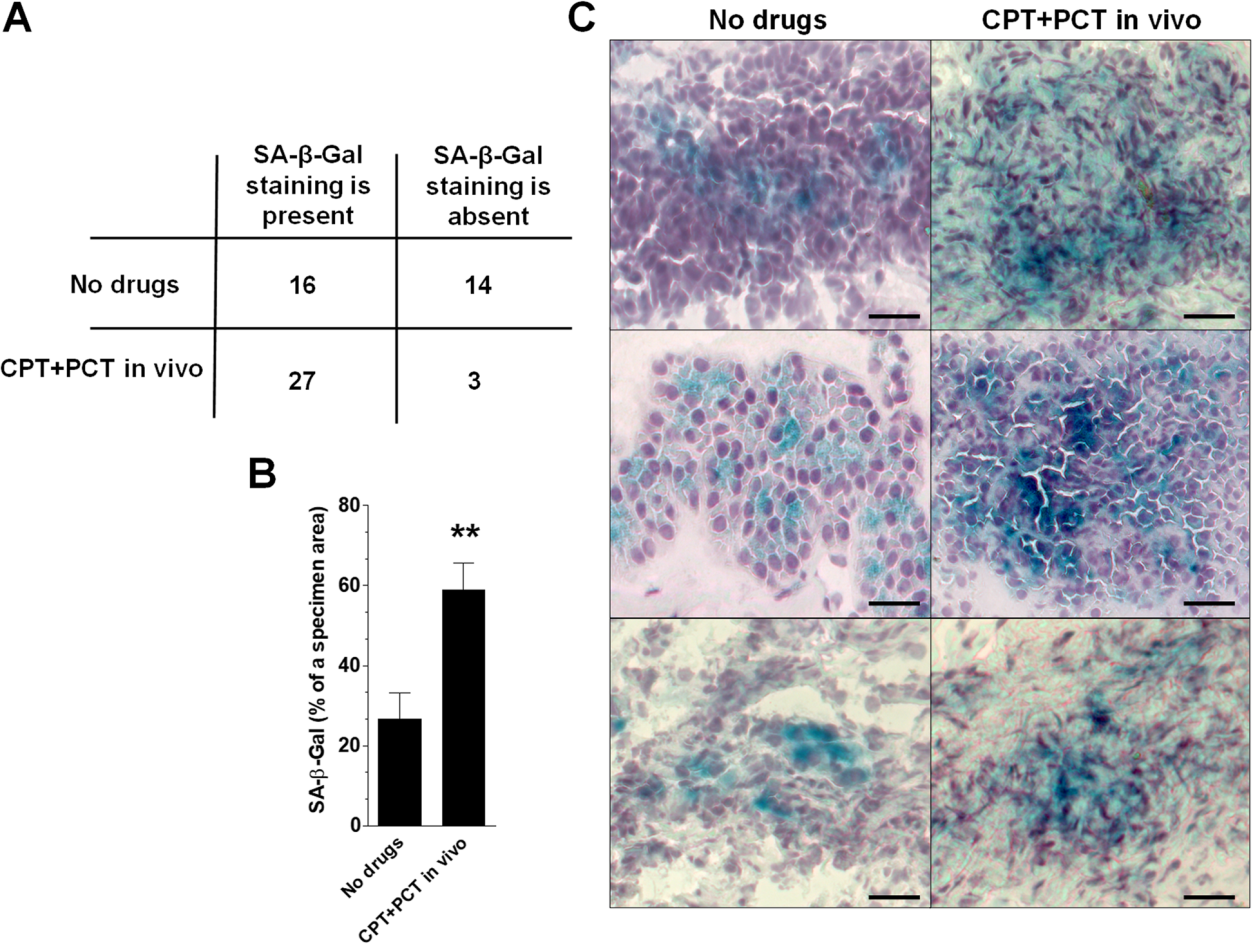
Presence of senescent, SA-β-Gal-marked ovarian cancer cells in tumors in vivo. Quantification of senescent cancer cell frequency in tumors from chemotherapy-naïve patients and from patients treated with CPT + PCT before cytoreduction (**A**). Determination of SA-β-Gal staining area in cancerous tissue within tumors from patients displaying signs of cellular senescence (**B**). Representative pictures demonstrating green SA-β-Gal staining in both groups of patients (**C**). The measurements were performed using tumors obtained from 30 different patients per group. The results depicted on panel B are expressed as the means ± SEM. ** *p* < 0.01 vs. No drugs. Magnification × 400; bar = 50 μm

Expression of SA-β-Gal, the commonly accepted biochemical marker of cellular senescence, was quantified in tumors from 30 chemotherapy-naïve patients with EOC and from 30 patients who received CPT and PCT prior cytoreduction. Each tumor was cut into 10 specimens which were subjected to cytochemical detection of the enzyme. We found that SA-β-Gal staining was present in 53% of patients who were not subjected to chemotherapy. For as many as 90% of patients treated with CPT and PCT we found SA-β-Gal-positively stained areas in at least 2 out of 10 specimens analyzed (Fig. 1A). Upon further analysis of every SA-β-Gal-positive tumor area we discovered that the magnitude of cellular senescence within cancerous tissue (Fig. 1B) and the intensity of the enzyme staining (Fig. 1C) were significantly higher in tumors from patients experienced chemotherapy.

### Molecular characteristics of drug-induced senescence in vitro and in vivo

In order to establish an in vitro model of drug-induced senescence of pEOCs which will resemble in vivo conditions in patients subjected to chemotherapy, we have recently created and optimized a regimen of cell exposure to CPT and PCT [11]. In this study, we compared a wide array of senescence-associated parameters between pEOCs whose senescence was induced by CPT + PCT in vitro and those derived from patients treated with CPT + PCT in vivo and for which senescence was triggered by serial passages in the absence of drugs in vitro. The results of this comparative analysis are shown in Table 1. It reveals that generally speaking all the tested parameters characterizing the course of senescence are equal between these two groups. This includes the percentages of cells bearing SA-β-Gal( +)/γ-H2A.X( +) phenotype, changes in the expression of cell cycle inhibitors (p16, p21, p53), lack of either telomere erosion or telomerase (hTERT) activity decline, non-telomeric localization of DNA damage foci, growth arrest in G2/M phase of cell cycle, and only sporadic apoptosis. Interestingly, however, young, early-passage pEOCs from patients who underwent chemotherapy in vivo were characterized by a significantly higher fraction of senescent [SA-β-Gal( +)/γ-H2A.X( +)] cells and cells expressing p16 cell cycle inhibitor compared with young cells from chemotherapy-naïve individuals.

**Table 1:** A comparison of senescence-associated parameters in pEOCs senesced upon their exposure to carboplatin (CPT) and paclitaxel (PCT) in vitro and in vivo

**Parameter (unit)**	n	**CPT + PCT in vitro**		**CPT + PCT in vivo**	
		**Young**	**Senescent**	**Young**	**Senescent**
SA-β-Gal( +)/γ-H2A.X( +) cells (%)	8	1 ± 1	73 ± 12	17 ± 2**	69 ± 10
p16-positive cells (%)	6	4 ± 2	62 ± 9	24 ± 6*	69 ± 7
p21-positive cells (%)	6	6 ± 1	42 ± 8	11 ± 8	54 ± 6
p53-positive cells (%)	6	4 ± 3	50 ± 6	7 ± 3	43 ± 5
telomere length (kbp)	6	4.4 ± 0.2	4.2 ± 0.1	4.4 ± 0.1	4.3 ± 0.1
telomerase activity (TPG)	6	2.1 ± 0.3	1.9 ± 0.3	1.8 ± 0.1	1.9 ± 0.2
γ-H2A.X-telomere colocalization (%)	10	6 ± 2	8 ± 4	6 ± 3	7 ± 1
G1 phase cells (%)	7	62 ± 4	48 ± 6	54 ± 8	41 ± 6
S phase cells (%)	7	21 ± 6	3 ± 5	19 ± 7	6 ± 1
G2/M phase cells (%)	7	17 ± 6	49 ± 1	27 ± 4	53 ± 6
subG1 cells (%)	6	0.8 ± 0.4	2.8 ± 3.4	1.5 ± 1.3	2.8 ± 1.2

Note that “Young” cells from CPT + PCT in vitro group were not treated with drugs neither in vitro nor in vivo. Experiments were performed using pEOC cultures obtained from different patients. The results are expressed as mean ± SEM

*TPG* Total Product Generated

^*^—*P* < 0.05 vs. in vitro; **—*P* < 0.01 vs. in vitro

### Senescence-associated secretory phenotype in cancer cells

Another functional feature of pEOCs which was analyzed and compared between spontaneous senescence of cancer cells, drug-induced senescence in vitro, and replicative senescence of cells from patients subjected to CPT + PCT in vivo was the development of senescence-associated secretory phenotype (SASP). To this end, 24 proteins engaged in a variety of aspects of cancer cell progression, such as: angiogenesis, ECM remodeling and invasion, inflammation, proliferation, and migration was quantified in conditioned media (CM) from young and senescent cells. The results we obtained showed that the SASP profile was the most intense with respect to spontaneously senescent pEOCs. For these cells, the secretion of ANG1, CXCL8/IL-8, FGF5, VEGF, ADAM12, PDGF-D, tPA, TGF-β1, TIMP-1, TSP-1, CCL2/MCP-1, ICAM-1, IL-6, VCAM-1, CCL11, CXCL12/SDF-1, EGF, HGF, IGF-1, and NRP-1 was significantly higher with respect to in vitro CPT + PCT-treated cells, senescent cells from patients subjected to CPT + PCT in vivo, or both (Table 2).

**Table 2:** Senescence-associated secretory phenotype in pEOCs undergoing spontaneous and drug-induced senescence in vitro and in vivo

		**No drugs (spontaneous)**		**CPT + PCT in vitro**	**CPT + PCT in vivo**	
**Process**	**Protein**	**young**	**senescent**	**senescent**	**young**	**senescent**
Angiogenesis	ANG1 (pg/10^5^ cells)	654 ± 316	5787 ± 1171^a^	2353 ± 356^a^*	1510 ± 343^a^	3186 ± 631^b^*
	bFGF (pg/10^5^ cells)	6 ± 1	44 ± 5^a^	49 ± 5^a^	5 ± 1	30 ± 8^b^
	CXCL8/IL-8 (pg/10^5^ cells)	129 ± 48	613 ± 54^a^	748 ± 142^a^	138 ± 61	414 ± 134^b^*
	FGF5 (fg/10^5^ cells)	839 ± 98	7034 ± 181^a^	2873 ± 89^a^*	1554 ± 163^a^	4982 ± 212^b^*
	VEGF (pg/10^5^ cells)	89 ± 18	228 ± 42^a^	132 ± 22^a^*	91 ± 35	283 ± 48^b^
ECM remodeling and invasion	ADAM12 (pg/10^5^ cells)	254 ± 63	2330 ± 562^a^	646 ± 81^a^*	802 ± 348^a^	1496 ± 255^b^*
	PDGF-D (fg/10^5^ cells)	625 ± 134	6695 ± 1354^a^	2411 ± 771^a^*	762 ± 235	4485 ± 622^b^*
	tPA (pg/10^5^ cells)	41 ± 9	343 ± 53^a^	282 ± 19 ^a^*	118 ± 26^a^	169 ± 13 ^b^*
	TGF-β1 (pg/10^5^ cells)	19 ± 6	107 ± 33^a^	29 ± 3*	12 ± 4	31 ± 9 ^b^*
	TIMP-1 (pg/10^5^ cells)	473 ± 127	3802 ± 592^a^	1613 ± 304^a^*	858 ± 346^a^	2702 ± 369^b^*
	TSP-1 (ng/10^5^ cells)	11 ± 2	93 ± 11^a^	39 ± 5^a^*	21 ± 4^a^	69 ± 8^b^*
	uPA (pg/10^5^ cells)	33 ± 9	104 ± 25^a^	78 ± 20	24 ± 4	104 ± 11^b^
Inflammation	CCL2/MCP-1 (pg/10^5^ cells)	130 ± 37	1131 ± 230^a^	470 ± 57^a^*	273 ± 28^a^	776 ± 90^b^*
	ICAM-1 (pg/10^5^ cells)	256 ± 48	830 ± 143^a^	607 ± 80^a^*	241 ± 38	694 ± 135
	IL-6 (pg/10^5^ cells)	25 ± 6	482 ± 154^a^	119 ± 7^a^*	65 ± 15^a^	449 ± 33^b^
	VCAM-1 (pg/10^5^ cells)	64 ± 16	285 ± 76^a^	144 ± 30^a^*	107 ± 25^a^	275 ± 63
Proliferation and migration	CCL11 (pg/10^5^ cells)	45 ± 12	364 ± 69^a^	148 ± 26^a^*	80 ± 33^a^	254 ± 32^b^
	CXCL1/GRO-1 (pg/10^5^ cells)	75 ± 11	497 ± 100^a^	572 ± 143^a^	204 ± 111^a^	532 ± 88^b^
	CXCL12/SDF-1 (pg/10^5^ cells)	2 ± 1	14 ± 5^a^	5 ± 1^a^*	3 ± 1	9 ± 2^b^
	CXCL5 (pg/10^5^ cells)	16 ± 4	37 ± 11^a^	149 ± 24^a^*	31 ± 7^a^	43 ± 15
	EGF (fg/10^5^ cells)	744 ± 23	4268 ± 199^a^	2831 ± 508^a^*	3175 ± 757^a^	5002 ± 449^b^
	HGF (pg/10^5^ cells)	30 ± 6	239 ± 22^a^	54 ± 14*	15 ± 3^a^	59 ± 6^b^*
	IGF-1 (pg/10^5^ cells)	15 ± 5	124 ± 28^a^	37 ± 3^a^*	12 ± 6	616 ± 179^b^*
	NRP-1 (pg/10^5^ cells)	62 ± 18	494 ± 86^a^	206 ± 34^a^*	114 ± 47^a^	358 ± 50^b^

Note that “young” cells in the “no drugs” group are common for “no drugs” senescent cells and “CPT + PCT in vitro senescent” cells (they originate from the same donor). Experiments were performed using pEOC cultures obtained from 8 different patients (in the “no drugs” and “CPT + PCT in vivo” groups, respectively). The results are expressed as mean ± SEM. ^a^ – *P* < 0.05 vs. young no drugs cells; ^b^ – *P* < 0.05 vs. young CPT + PCT in vivo cell; *—*P* < 0.05 vs. senescent no drugs cells

Interesting effect was found regarding ANG1, FGF5, ADAM12, tPA, TIMP-1, TSP-1, CCL2/MCP-1, IL-6, VCAM-1, CCL11, CXCL1/GRO-1, CXCL5, EGF, and NRP-1 whose baseline production by young cells from patients undergoing chemotherapy in vivo was significantly higher than for young cells from chemotherapy-naïve donors.

### Senescent cancer cell-driven progression of non-senescent cancer cells in vitro

**Fig. 2:**
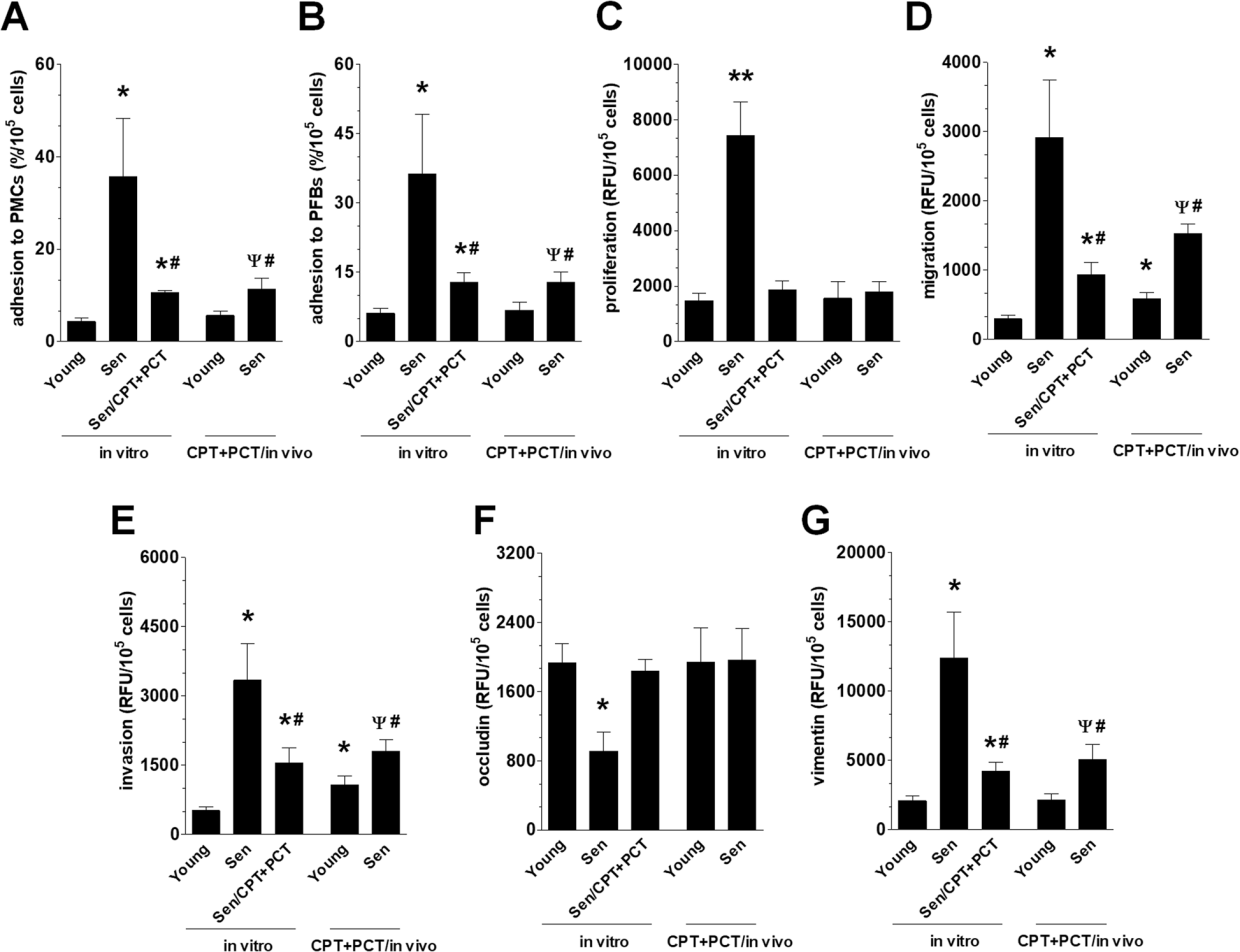
Effect of senescent pEOCs secretome on ovarian cancer cell progression in vitro. Analysis of pEOCs-derived conditioned medium effect on adhesion (to PMCs – **A** and to PFBs—**B**) proliferation (**C**), migration (**D**), and invasion (**D**) of non-senescent ovarian cancer cells. Quantification of occludin (**F**) and vimentin (**G**) expression as markers of EMT. Results derive from 6–8 independent experiments with pEOCs obtained from different donors. The results are expressed as the means ± SEM. * *p* < 0.05; ** *p* < 0.01 vs. “Young in vitro” cells; ^#^ *p* < 0.05 vs. “Sen in vitro” cells; ^Ψ^ *p* < 0.05 vs. “Young/CPT + PCT in vivo” cells. RFU – Relative Fluorescence Units

Three models of pEOCs senescence were compared regarding the ability their autologous CM to promote vital elements of cancer cell expansion, that is adhesion, proliferation migration, invasion, and epithelial-mesenchymal transition (EMT). Our comparative analysis of proliferating (non-senescent) pEOCs adhesion to normal peritoneal mesothelial cells (PMCs) and peritoneal fibroblasts (PFBs) revealed that CM derived from all types of senescent pEOCs stimulates their attachment to normal cells more effectively than CM from young cells, albeit the strongest effect was recorded in both cases for CM generated by spontaneously senescent pEOCs (Fig. 2A, B). As per cell proliferation, only CM from spontaneously senescent pEOCs was able to promote this process more than the secretome of young cells (Fig. 2C). The migration (towards the chemotactic activity of CM) and invasion (towards CM through the Basement Membrane Extract) of cancer cells were also fueled by all kinds of senescent pEOCs, however, similarly to adhesion, the strongest stimulation was displayed by spontaneously senescent cells (Fig. 2D, E). The efficacy of migration and invasion of cancer cells upon exposure to CM from young cells obtained from chemotherapy-subjected patients was significantly stronger than CM from chemotherapy-naïve individuals. Last but not least, CM from spontaneously senescent cells was the sole capable of decreasing the expression of occluding (a negative marker of EMT). At the same time, CMs from all types of senescent cells increased the expression of vimentin (a positive marker of EMT), however, the activity of CM from spontaneously senescent cells was the most pronounced (Fig. 2F, G).

### Pro-angiogenic activity of senescent cancer cells

**Fig. 3:**
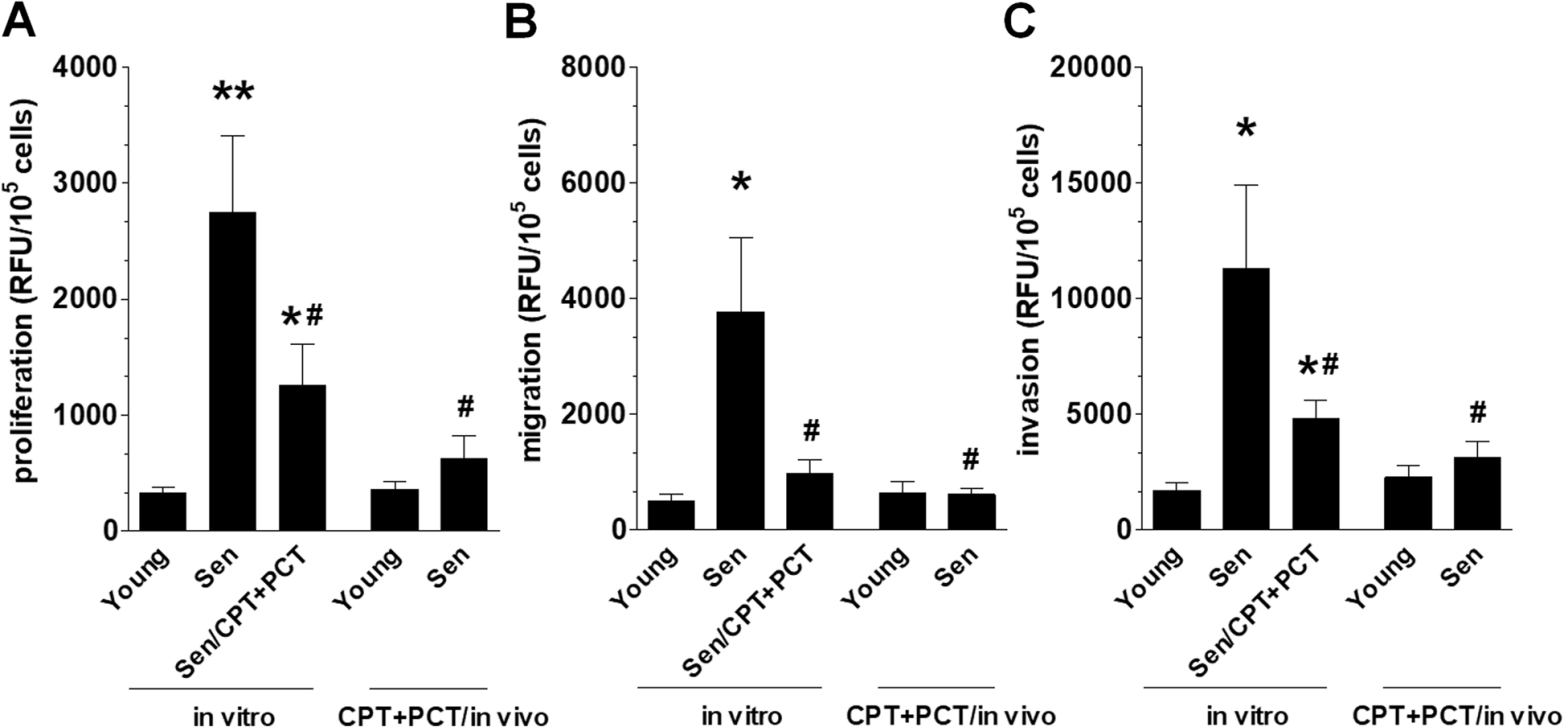
Effect of senescent pEOCs secretome on angiogenic behavior of vascular endothelial cells in vitro. Analysis of pEOCs-derived conditioned medium effect on proliferation (**A**), migration (**B**), and invasion (**C**) of vascular endothelial cells (HUVECs). Results derive from 6–8 independent experiments with pEOCs obtained from different donors. The results are expressed as the means ± SEM. * *p* < 0.05; ** *p* < 0.01 vs. “Young in vitro” cells; ^#^ *p* < 0.05 vs. “Sen in vitro” cells. RFU – Relative Fluorescence Units

Three angiogenic reactions of vascular endothelial cells (HUVECs), that is proliferation, migration, and invasion were tested in response to CM generated by young and senescent pEOCs. Proliferation of HUVECs was stimulated by all three types of senescent pEOCs but the effect exerted by spontaneously senescent cells was the greatest (Fig. 3A). Migration of endothelial cells was supported exclusively by spontaneously senescent cells (Fig. 3B), whereas invasion was promoted by CM from spontaneously senescent cells and those for which senescence was elicited by CPT + PCT in vitro, with the strongest effect on the side of the spontaneously senescent cells (Fig. 3C).

### Senescence-related functional characteristics of normal peritoneal cells subjected to senescent cancer cells

**Fig. 4:**
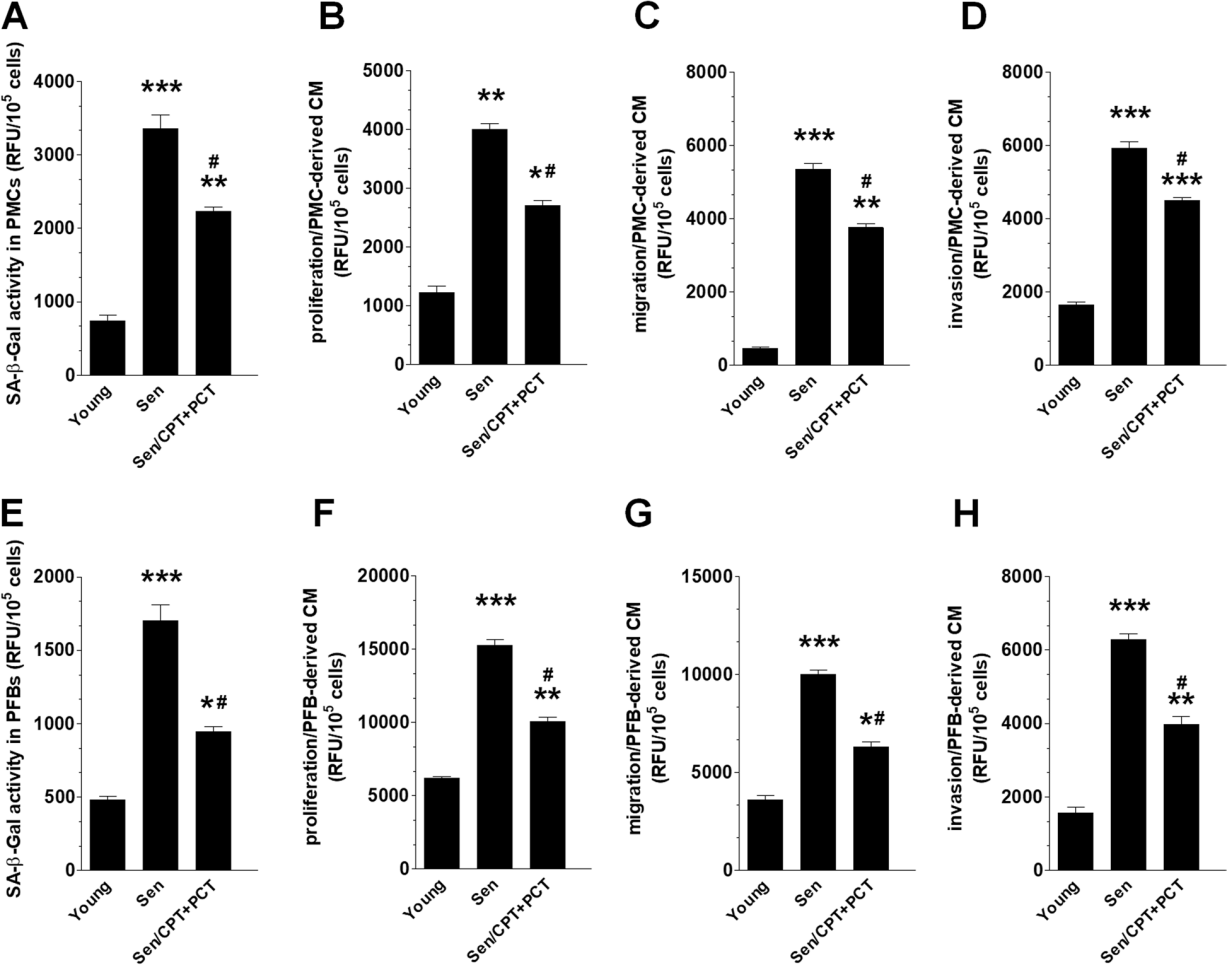
Paracrine effects of senescent pEOCs on senescence and pro-cancerogenic activity of normal PMCs (**A**-**D**) and PFBs (**E**–**H**). Quantification of SA-β-Gal activity in PMCs and PFBs exposed to pEOCs-derived conditioned medium (**A**, **E**). Proliferation (**B**, **F**), migration (**C**, **G**), and invasion (**D**, **H**) of non-senescent ovarian cancer cells in response to autologous PMCs- and PFBs-derived conditioned medium upon their preexposure to conditioned medium generated by young and senescent pEOCs. The experiments were performed using pooled PMCs and PFBs from 6 different donors and ovarian cancer cells from 6 different patients. The results are expressed as mean ± SEM. The results are expressed as the means ± SEM. * *p* < 0.05; ** *p* < 0.01; *** *p* < 0.001 vs. “Young” cells; ^#^ *p* < 0.05 vs. “Sen” cells. RFU – Relative Fluorescence Units

CMs from spontaneous and drug-inducible (in vitro) pEOCs were applied to young PMCs and PFBs to determine the induction of their senescence and the capacity of CM generated by normal cells under such conditions to support cancer cell proliferation, migration, and invasion. The quantification of SA-β-Gal-dependent fluorescence revealed that both types of senescent pEOCs induce the enzyme in PMCs and PFBs, albeit the most robust induction of senescence was exerted in both cases by CM from spontaneously senescent cells (Fig. 4A, E). Subsequently, CM generated by PMCs and PFBs exposed to CM from senescent pEOCs stimulated proliferation (Fig. 4B, F), migration (Fig. 4C, G), and invasion (Fig. 4D, H) of non-senescent ovarian cancer cells. For all the three tested phenomena, the effects related to spontaneously senescent pEOCs were considerably stronger.

### Secretory properties of normal peritoneal cells subjected to senescent cancer cells

Taking into account the fact that senescent pEOCs induce SA-β-Gal in normal PMCs and PFBs, and that senescent cells typically display SASP, 19 proteins relevant for cancer cells progression was quantified in PMCs- and PFBs-derived CM upon their pre-incubation with CM generated by young and senescent pEOCs. For PMCs, all the tested proteins were hypersecreted by senescent cells, irrespective of the type of senescence. In addition, the release of 8 out of 19 proteins (ANG1, CXCL8, VEGF, PDGF-D, tPA, TGF-β1, IL-6, VCAM-1) was significantly higher in response to CM from spontaneously senescent pEOCs, the release of 9 molecules was comparable in both groups, and the release of 2 proteins (CXCL12/SDF-1 and NRP-1) was higher in response to drug-inducible senescent pEOCs (Table 3). As per PFBs’ secretome, all but two proteins (FGF5, CCL11) were hypersecreted upon treatment with CM from senescent pEOCs. The release of 12 out of 17 remaining up-regulated molecules was higher in response to CM from spontaneously senescent pEOCs, and the release of CXCL-8/IL-8, VEGF, PDGF-D, tPA, and TGF-β1 by spontaneously senescent and drug-inducible senescent cells was comparable (Table 4).

**Table 3:** Secretory phenotype of peritoneal mesothelial cells subjected to a conditioned medium generated by young and senescent ovarian cancer cells

		**No drugs (spontaneous)**		**CPT + PCT in vitro**
**Process**	**Protein**	**young**	**senescent**	**senescent**
Angiogenesis	ANG1 (pg/10^5^ cells)	87 ± 8	245 ± 12^a^	192 ± 10^a^*
	CXCL8/IL-8 (pg/10^5^ cells)	53 ± 4	104 ± 6^a^	79 ± 2^a^*
	FGF5 (pg/10^5^ cells)	18 ± 3	41 ± 3^a^	43 ± 3^a^
	VEGF (pg/10^5^ cells)	66 ± 1	579 ± 50^a^	351 ± 29^a^*
ECM remodeling and invasion	ADAM12 (pg/10^5^ cells)	12 ± 1	46 ± 7^a^	39 ± 4^a^
	PDGF-D (fg/10^5^ cells)	46 ± 5	173 ± 17^a^	122 ± 4^a^*
	tPA (pg/10^5^ cells)	7 ± 1	38 ± 4^a^	20 ± 2 ^a^*
	TGF-β1 (pg/10^5^ cells)	61 ± 5	366 ± 32^a^	261 ± 17^a^*
	TIMP-1 (pg/10^5^ cells)	69 ± 3	193 ± 12^a^	185 ± 10^a^
	uPA (pg/10^5^ cells)	18 ± 1	50 ± 2^a^	48 ± 4^a^
Inflammation	CCL2/MCP-1 (pg/10^5^ cells)	16 ± 1	60 ± 13^a^	75 ± 7^a^
	ICAM-1 (pg/10^5^ cells)	7 ± 1	38 ± 5^a^	35 ± 2^a^
	IL-6 (pg/10^5^ cells)	65 ± 4	463 ± 44^a^	274 ± 5^a^*
	VCAM-1 (pg/10^5^ cells)	46 ± 2	104 ± 10^a^	76 ± 7^a^*
Proliferation and migration	CCL11 (pg/10^5^ cells)	4 ± 1	13 ± 1^a^	12 ± 1^a^
	CXCL1/GRO-1 (pg/10^5^ cells)	16 ± 1	75 ± 7^a^	64 ± 9^a^
	CXCL12/SDF-1 (pg/10^5^ cells)	83 ± 5	101 ± 5^a^	178 ± 8^a^*
	IGF-1 (pg/10^5^ cells)	10 ± 1	45 ± 5^a^	47 ± 7^a^
	NRP-1 (pg/10^5^ cells)	72 ± 5	112 ± 16^a^	184 ± 2^a^*

Note that “young” cells in the “no drugs” group are common for “no drugs” senescent cells and “CPT + PCT in vitro senescent” cells (they originate from the same donor). Experiments were performed using mesothelial cells (pooled) and ovarian cancer cells from 6 different patients. The results are expressed as mean ± SEM. ^a^ – *P* < 0.05 vs. young no drugs cells; *—*P* < 0.05 vs. senescent no drugs cells

**Table 4:** Secretory phenotype of peritoneal fibroblasts subjected to conditioned medium generated by young and senescent ovarian cancer cells

		**No drugs (spontaneous)**		**CPT + PCT in vitro**
**Process**	**Protein**	**young**	**senescent**	**senescent**
Angiogenesis	ANG1 (pg/10^5^ cells)	46 ± 13	301 ± 17^a^	140 ± 7^a^*
	CXCL8/IL-8 (pg/10^5^ cells)	120 ± 4	274 ± 26^a^	231 ± 10^a^
	FGF5 (pg/10^5^ cells)	43 ± 8	58 ± 9	53 ± 4
	VEGF (pg/10^5^ cells)	27 ± 2	37 ± 5^a^	44 ± 6^a^
ECM remodeling and invasion	ADAM12 (pg/10^5^ cells)	52 ± 3	76 ± 2^a^	54 ± 2*
	PDGF-D (fg/10^5^ cells)	174 ± 16	252 ± 23^a^	222 ± 20^a^
	tPA (pg/10^5^ cells)	18 ± 1	50 ± 4^a^	49 ± 7^a^
	TGF-β1 (pg/10^5^ cells)	22 ± 4	52 ± 5^a^	45 ± 2^a^
	TIMP-1 (pg/10^5^ cells)	149 ± 5	250 ± 8^a^	165 ± 5^a^*
	uPA (pg/10^5^ cells)	41 ± 1	77 ± 4^a^	49 ± 4*
Inflammation	CCL2/MCP-1 (pg/10^5^ cells)	75 ± 6	117 ± 18^a^	77 ± 7^a^*
	ICAM-1 (pg/10^5^ cells)	15 ± 2	64 ± 3^a^	39 ± 2^a^*
	IL-6 (pg/10^5^ cells)	50 ± 8	742 ± 82^a^	246 ± 14^a^*
	VCAM-1 (pg/10^5^ cells)	104 ± 4	220 ± 17^a^	114 ± 9^a^*
Proliferation and migration	CCL11 (pg/10^5^ cells)	8 ± 2	11 ± 2	9 ± 3
	CXCL1/GRO-1 (pg/10^5^ cells)	110 ± 3	186 ± 14^a^	121 ± 11*
	CXCL12/SDF-1 (pg/10^5^ cells)	99 ± 3	184 ± 9^a^	120 ± 8^a^*
	IGF-1 (pg/10^5^ cells)	33 ± 4	56 ± 6^a^	41 ± 2^a^*
	NRP-1 (pg/10^5^ cells)	40 ± 6	371 ± 41^a^	66 ± 3^a^*

Note that “young” cells in the “no drugs” group are common for “no drugs” senescent cells and “CPT + PCT in vitro senescent” cells (they originate from the same donor). Experiments were performed using fibroblasts (pooled) and ovarian cancer cells from 6 different patients. The results are expressed as mean ± SEM. ^a^ – *P* < 0.05 vs. young no drugs cells; *—*P* < 0.05 vs. senescent no drugs cells

### Senescence of ovarian cancer cells and tumor growth in vivo

**Fig. 5:**
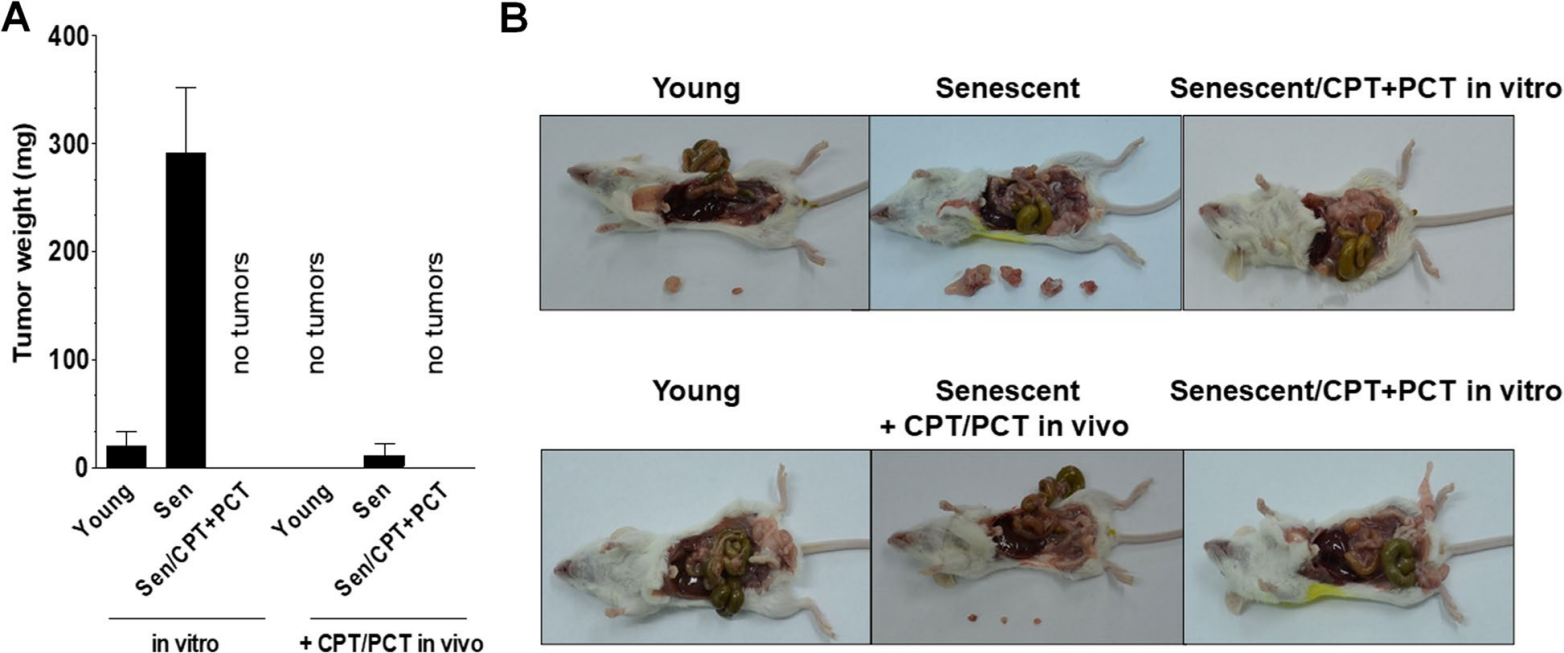
The influence of senescent pEOCs on ovarian tumor development in mouse peritoneal cavity in vivo. Comparison of the total weight of tumors that developed intraperitoneally 21 days after the implantation of young or senescent + young pEOCs (1:1 ratio) (**A**). Representative pictures of animals and excised tumors from each group (**B**)

The immunocompromised Scid mice were used to generate xenografts at the i.p. injection of young pEOCs, spontaneously senescent, and drug-inducible senescent cells. Senescent cells were mixed 1:1 with young cells. At the end of the 21-day experiment, tumors developed in mice injected with young and spontaneously senescent cells, albeit in the latter group the incidence (5/5 animals vs. 2/5 animals) and the total weight of tumors were significantly higher (Fig. 5). The experiment also included three other groups of animals in which CPT + PCT were administered in vivo (every 3 days) with an injection of young and senescent pEOCs. In those animals, chemotherapy inhibited the tumor growth either in young pEOCs or spontaneously senescent pEOCs xenografts (Fig. 5).

### Senescence of ovarian cancer cells and the expression of genes responsible for drug resistance

Expression of 10 genes associated according to literature with cancer cell resistance to platins and taxanes was investigated at mRNA level using qPCR. These included *ABCB1/MDR1, ABCC4, AKT1, PLK2, BIRC5/Survivin, CHEK1, CHEK2, PHB1/Prohibitin-1, PHB2/Prohibitin-2, and CCND1/cyclin D1.* An analysis of these transcripts showed that senescence of pEOCs, irrespective of the kind, is not associated with any changes in the expression of the tested genes (Table 5).

**Table 5:** Expression of mRNA for genes associated with drug-resistance in young and senescent pEOCs

Gene	**No drugs (spontaneous)**		**CPT + PCT in vitro**
	**young**	**senescent**	**senescent**
*ABCB1/MDR1*	1.4 ± 0.4	1.8 ± 0.7	1.3 ± 0.7
*ABCC4*	1.2 ± 0.3	1.1 ± 0.6	1.4 ± 0.6
*AKT1*	1.1 ± 0.2	1.9 ± 0.9	1.6 ± 0.7
*PLK2*	1.2 ± 0.3	2.8 ± 1.4	1.3 ± 0.6
*BIRC5/Survivin*	1.2 ± 0.3	0.9 ± 0.3	1.2 ± 0.5
*CHEK1*	1.7 ± 0.8	2.1 ± 1.0	1.2 ± 0.7
*CHEK2*	1.8 ± 0.8	2.2 ± 1.5	1.0 ± 0.4
*PHB1/Prohibitin-1*	2.5 ± 1.2	1.9 ± 0.9	2.8 ± 2.2
*PHB2/Prohibitin-2*	1.4 ± 0.5	1.0 ± 0.5	1.0 ± 0.6
*CCND1/cyclin D1*	1.1 ± 0.3	2.2 ± 1.0	1.0 ± 0.6

Experiments were performed using pEOCs obtained from 6 different patients. Each sample was tested in duplicate. The results are expressed as mean ± SEM. Expression of mRNA for β-actin was used as the reference

## Discussion

This study is the first in which pro-cancerogenic outcomes of spontaneous and drug-induced senescence of cancer cells (here: pEOCs) were demonstrated and parallelly compared in vitro and in vivo. Previously, the comparisons between cells undergoing replicative and stress-induced premature senescence (SIPS), concentrated on either genetic signatures [13] and proteomic profiles [14], were made exclusively on normal cells, particularly fibroblasts. In another elegant study, also performed on normal cells, specifically vascular smooth muscle cells, Bielak-Zmijewska and colleagues compared replicatively senescent cells and cells forced to senescence by doxorubicin, and demonstrated some mechanistic differences in both these processes [15]. It should be stressed, however, that the comparison of direct and indirect (normal cell-dependent) pro-cancerogenic activities of spontaneously senescent and drug-inducible senescent cancer cells has never been made before, which makes leading the narrative axis of our results discussion somehow difficult.

Senescent pEOCs are present in tumors from chemotherapy-naïve and CPT + PCT-treated patients, however in the latter they are more frequent and occupy a larger tumor area. This difference was, to some extent, predictable as a genetic insult experienced by pEOCs exposed to the chemotherapeutics (SIPS) is surely greater and more senescence-promoting than a time-consuming, progressive, divisions-driven, and environmental cytokine supported spontaneous senescence of these cells [10]. In this context, it can be recalled that both carboplatin [16] and paclitaxel [17] cause severe DNA damage to cells, which is widely considered as a primary cause of cellular senescence [18].

Senescence-determining events in pEOCs from CPT + PCT-treated patients occur at a higher rate than those in spontaneously senescing cells, which confirms a higher expression of senescence biomarkers (SA-β-Gal( +)/γ-H2A.X( +)/p16( +) phenotype) and an elevated secretion of some proteins (an equivalent of SASP) in cells directly transferred from tumors in vivo to culture conditions in vitro. A similar prevalence of expression of senescence biomarkers in cells undergoing drug-dependent senescence over replicatively senescent cells has been described for SA-β-Gal staining in vascular smooth muscle cells [15]. At the same time, these observations support previous reports showing that features of enhanced senescence occurring in vivo can be transferred to culture conditions and manifested in the phenotype of early-passage cells [19, 20].

The core of this study was to verify whether senescent pEOCs may stimulate — basically through the products of their secretome — their non-senescent counterparts progression analogically to a cancer-promoting effect repeatedly described for various types of normal senescent cells [4, 21]. Surprisingly, our results showed that more robust senescence of pEOCs in vivo did not translate into a pro-cancerogenic potential of these cells. A direct comparison of spontaneously and drug-inducible senescent pEOCs secretome revealed that the former stimulate progression of non-senescent pEOCs, angiogenic reactions of vascular endothelium, and senescence-dependent pro-cancerous effects of normal PMCs and PFBs far more efficiently than their drug-dependent counterparts. In other words, although chemotherapeutics clearly exacerbated senescence of pEOCs in vivo and in vitro, the effect of this process on cancer cell progression was lower than the effect of cells whose senescence was on a smaller scale. The differences in both senescence mechanisms reported previously [10, 11] are, however, an unlikely explanation for the observed pro-cancerogenic outcomes.

At the same time, the prominent tumor-promoting activity of spontaneously senescent cells could be explained by a distinct pattern of SASP profiles that developed in both kinds of analyzed cells. Namely, senescent cells from chemotherapy-naïve patients secreted to environment significantly higher amounts of several soluble mediators of cancer spread. These included, among others, VEGF—known to promote ovarian cancer neovascularization [22], TGF-β1—recognized as a mediator of EMT [23] and increased ovarian cancer cell invasion [24], proinflammatory and ovarian cancer cell growth-promoting IL-6 [25], and pro-migratory HGF [26]. Moreover, spontaneously senescent pEOCs appeared to be able to reprogram the secretory capacity of normal peritoneal cells, e.g. by up-regulating the release by PMCs of PDGF-D that modulates extracellular matrix remodeling and stimulates ovarian cancer cell invasion [27] or by PFBs of NRP-1 and CXCL12/SDF-1 fueling ovarian cancer motility and metastatic potential [28, 29]. Intriguingly, a similar reprogramming of secretory properties has previously been found in the opposite direction. Namely, senescent PMCs were able to induce the pro-angiogenic phenotype in ovarian cancer cells in IL-6/TGF-β1-dependent mechanism [30]. This similarity strengthens the view that normal peritoneal cells and ovarian cancer cells remain in a highly dynamic state of interactions, in which senescence of both groups of cells seems to act as a trigger of various pro-tumoral activities.

The dominance of spontaneous over drug-induced senescence with respect to cancer progression in vitro was finally confirmed in vivo, where only spontaneously senescent cells were able to increase the development of peritoneal xenografts, fortunately without causing their increased drug resistance.

## Conclusions

Our findings imply that senescence of pEOCs, and especially the spontaneous variant of this process, should be treated by oncologists as independent pathogenetic factor in disease progression. Given the potential for senescent cell elimination or their SASP-dependent activity inhibition presented by senolytics and senostatics, respectively, the implementation of such drugs into standard EOC therapy should be considered [31].

## Methods

### Materials

Unless otherwise stated, all chemicals and plastics were obtained from Sigma (St. Louis, MO) and Nunc (Roskilde, Denmark), respectively.

### Patients

The study was based on tumors obtained from 60 patients with serous ovarian cancer (stage III or IV according to the criteria of the International Federation of Gynecology and Obstetrics). One half of them were chemotherapy-naïve whereas the other half received CPT and PCT prior cytoreduction [32]. The patients were between 36 and 88 years old. The tumors were fixed in 4% formalin, embedded in paraffin, and cut into 3 μm sections. Deparaffinization, rehydration and epitope retrieval were performed using Envision Flex Target Retrieval Solution (Dako, Glostrup, Denmark). The cancerous nature of the tissues was identified using standard H + E staining by a pathomorphologist. Cytochemical detection of senescence-associated β-galactosidase (SA-β-Gal) was conducted following methodology described by Dimri et al. [33] and planimetric analyses of a green-stained area reflecting the presence of SA-β-Gal-positive cancer cells were performed as described in [10].

### Cells

Primary epithelial ovarian cancer cells (pEOCs) were isolated from tumors obtained during cytoreductive surgery. The methodology of cell isolation and identification was previously described in [10]. The cells were maintained in RPMI 1640 enriched in with L-glutamine (2 mM) and 20% FBS. Primary human peritoneal mesothelial cells (PMCs) and peritoneal fibroblasts (PFBs) were isolated by enzymatic dissagregation of the omentum, obtained from 14 patients undergoing abdominal surgery. PMCs were propagated in M199 medium supplemented with L-glutamine (2 mM), penicillin (100 U/mL), streptomycin (100 µg/mL), hydrocortisone (0.4 µg/mL) and 10% FBS, whereas PFBs were grown in Ham’s Nutrient Mixture F-12 medium supplemented similarly as the medium for PMCs. Human umbilical vein endothelial cells (HUVECs) were obtained from Lonza (Walkersville, MD, USA) and cultured in EBM™-2 Basal Medium with EGM™-2 SingleQuots™ Supplements (Lonza).

### Experimental conditions

pEOCs were forced to replicative senescence by serial passaging at fixed seeding density and time intervals, until complete exhaustion of their capacity to replicate [10]. Drug-induced senescence of pEOCs was reached by an exposure of 1^st^ passage cells to 50 µM CPT combined with 25 nM PCT (both from Cayman Chemical, Ann Arbor, MI, USA) and further procedures, exactly as described in [11]. In both regimens, senescence was confirmed by cell hypertrophic appearance, long-term inability to proliferate, and the presence of senescence biomarkers. As per terminology used in the study, spontaneous senescence of pEOCs refers to the state reached by cells from chemotherapy-naïve patients in response to their serial passaging in vitro. Drug-inducible senescence refers to the state reached by cells treated by CPT + PCT in vitro, as well as to cells from patients subjected to chemotherapy in vivo that lost their proliferative capacity after serial sub-cultivations in the absence of drugs in vitro.

### Biomarkers of cell growth and senescence

Colocalization of SA-β-Gal and the phosphorylated variant of histone H2A.X (γ-H2A.X) was performed according to cytochemical detection of the enzyme combined with a fluorescence-based method of γ-H2A.X foci detection, as described in [33, 34]. Activity of SA-β-Gal was determined using a fluorescence-based measurement of the rate of conversion of 4-methylumbelliferyl-β-D-galactopyranose (MUG) to 4-methylumbelliferone (4-MU), as described by Gary and Kindell [35]. Expression of p16, p21, and p53 cell cycle inhibitors was quantified using immunofluorescence, as described in [10]. Telomere length was evaluated using Absolute Human Telomere Length Quantification qPCR Assay Kit (ScienCell, Carlsbad, CA, USA) and telomerase (hTERT) activity using the TRAPEZE XL Telomerase Detection Kit (Merck, Darmstadt, Germany), both essentially as described in manufacturer’s recommendations. Analysis of telomeric localization of γ-H2A.X foci was conducted using the Telomere PNA FISH Kit/Cy3 (Dako, Carpinteria, CA, USA), as per manufacturer’s instructions. Cell distribution in particular phases of the cell cycle was examined using flow cytometry with propidium iodide-stained cells, as described in [36]. An intensity of apoptosis, determined according to the size of subG1 cell fraction displaying fragmented, low-molecular weight DNA, was estimated using flow cytometry, according to methodology described in [37].

### Functional tests

Adhesion of calcein-AM-probed (Molecular Probes, Invitrogen, Eugene, OR, USA) cancer cells to PMCs or PFBs was performed as described in [38]. Cell proliferation was tested in a 24-h protocol using Cell Proliferation Kit I (PromoKine; Heidelberg, Germany), as per manufacturer’s instructions. Migration of cells towards a conditioned medium used as a chemoattractant (for 4 h) was determined using ChemoTx migration chambers (Neuro Probe, Gaithersburg, MD, USA), as per manufacturer’s instructions. Cancer cell invasion was measured with a Cultrex 96 Well BME Cell Invasion Assay (Trevigen Inc., Gaithersburg, MD), as described in [39]. To evaluate epithelial-mesenchymal transition (EMT), expression of occludin (a marker of the epithelial phenotype) and vimentin (a marker of the mesenchymal phenotype) was quantified using immunofluorescence-based methods, essentially as described in [39, 40]. During the functional tests, behavior of non-senescent cancer cells, endothelial cells, and normal PMCs and PFBs was investigated upon their exposure to conditioned medium (CM) generated by young and senescent pEOCs in the presence of reduced (from 20 to 5%) FBS.

### Cell secretome

To generate samples of CM for cell secretome measurements, pEOCs were subjected to serum-free medium for 72 h. The samples of CM were then filtered and frozen at –80 °C until required. Concentrations of ANG1, bFGF, CXCL8/IL-8, VEGF, ADAM12, PDGF-D, tPA, TGF-β1, TIMP-1, TSP-1, uPA, CCL2/MCP-1, ICAM-1, IL-6, VCAM-1, CCL11, CXCL1/GRO-1, CXCL12/SDF-1, CXCL5, EGF, HGF, IGF-1, and NRP-1 were quantified using appropriate DuoSet® Immunoassay Development kits (R&D Systems, according to the manufacturer’s instructions. FGF5 was quantified using an assay purchased from ABCbiolab (Anaheim, CA, USA).

### Drug-resistance genes

Cells were lysed using the CellAmp Direct RNA Prep Kit for RT-PCR (Takara Bio, Otsu, Japan). Quantitative PCR was performed using the Takara PrimeScript RT Master MIX (Perfect Real Time) (Takara Bio) with the Fast Start Essential DNA Green Master (Roche, Basel, Switzerland) and appropriate primers (Sino Biological Inc., China). All reactions were performed on Light Cycler 480 (Roche) under following conditions: 5 min at 95 °C, 45 cycles of 10 s at 95 °C, and 1 min at 65 °C. The quality of each amplicon was evaluated on the basis of the course of dissociation curves obtained via Melt Curve Stage at the end of each PCR. The relative expression levels were determined according to the 2^−ΔΔCt^ method with the β-actin (*ACTB*) gene as the reference. The measurements included: ABCB1/MDR1 (cat. no. HP101566), ABCC4 (cat. no. HP104135), AKT1 (cat. no. HP100902), PLK2 (cat. no. HP101256), BIRC5/Survivin (cat. no. HP100392), CHEK1 (cat. no. HP100555), CHEK2 (cat. no. HP101292), PHB1/Prohibitin-1 (cat. no. HP101569), PHB2/Prohibitin-2 (cat. no. HP104907), and CCND1/cyclin D1 (cat. no. HP100789), and ACTB (cat. no. HP100001).

### In vivo experiments

Experiments were performed on 5 week old immunocompromised Scid mice (CB17/ I cr-Prkdc/I crI coCrl; Charles River, Wilmington, MA, USA). The animals were housed in individually ventilated cages (5/cage). After a quarantine period, the animals were injected i.p. with 2 × 10^6^ of young pEOCs or with a mixture of young pEOCs with senescent pEOCs (1:1 ratio) in 100 µl of sterile PBS. Health and welfare of the animals, including the progression of the intraperitoneal tumors was inspected every day. Starting from day 3 after cell implantation, CPT (80 mg/kg) and PCT (5 mg/kg) were administered i.p. to some animals every 3 days. The experiment was terminated after 21 days by animal scarification using carbon dioxide intoxication. After the euthanasia, the peritoneal cavity of experimental animals was inspected and tumors that developed in the peritoneum were excised, counted, weighed, and photographed.

### Statistics

Statistical analysis was performed using GraphPad Prism™ 6.00 software (GraphPad Software, San Diego, USA). The means were compared with repeated ANOVA with a post hoc Newman-Keuls test. The results are expressed as the mean ± SEM. Differences with a *p* < 0.05 were considered to be statistically significant.

## Data Availability

All data generated or analysed during this study are included in this published article.
